# Increasing obstructive sleep apnea risk is associated with albuminuria in Korean adults: cross-sectional analysis

**DOI:** 10.1038/s41598-024-57394-3

**Published:** 2024-03-20

**Authors:** Suk Won Chang, Ju Wan Kang

**Affiliations:** 1 Department of Otorhinolaryngology, Jeju National University College of Medicine, Jeju, Korea; 2 Department of Otorhinolaryngology, Jeju National University Hospital, Jeju, Korea; 3 Department of Otorhinolaryngology, Yongin Severance Hospital, Yonsei University College of Medicine, 363, Dongbaekjukjeon-Daero, Giheung-gu, Yongin, 16995 Korea

**Keywords:** Albuminuria, Obstructive sleep apnea, Albumin/creatinine ratio, Korean, Chronic kidney disease, Sleep disorders

## Abstract

Several studies have shown an association between albuminuria and obstructive sleep apnea (OSA). However, studies on the relationship between the STOP-BANG questionnaire that can screen for OSA and microalbuminuria are still insufficient. Therefore, this study attempted to clarify the relationship between microalbuminuria and OSA risk using the STOP-BANG questionnaire in Korean adults. A total of 7478 participants (3289 men and 4189 women) aged over 40 were enrolled in the Korean National Health and Nutrition Examination Survey from 2019 to 2020. STOP-BANG questionnaire to screen OSA was obtained from subjects. The urinary albumin/creatinine ratio (ACR) and proteinuria were measured via a single dipstick to evaluate renal function. The high OSA risk group had a higher mean ACR value than the low OSA risk group (36.8 ± 172.2 vs 17.7 ± 82.5; *P* < 0.001). The proportion of subjects with values of 30 ≤ ACR < 300 mg/g (11.9% vs 6.1%; *P* < 0.001) and ACR > 300 mg/g (2.1% vs 0.7%; *P* < 0.001) was significantly higher in high OSA risk group. Multivariate logistic regression results confirmed that microalbuminuria (OR 1.279, 95% confidence interval (CI) 1.068–1.532, *P* = 0.008) was significantly correlated with high OSA risk. In addition, significant correlation with high OSA risk was also found in macroalbuminuria (OR 1.684, 95% CI 1.073–2.530, *P* = 0.022) and proteinuria (OR 1.355, 95% CI 1.030–1.783, *P* = 0.030). We confirmed a significant correlation between high OSA risk and albuminuria/proteinuria in Korean adults. Therefore, renal function evaluation is required in high OSA risk patients, and OSA diagnosis through PSG test and treatment is necessary.

## Introduction

Obstructive sleep apnea (OSA) is a disease characterized by upper airway obstruction during sleep. Approximately 936 million adults worldwide have mild to severe OSA^1^. OSA is associated with obesity, metabolic syndrome, and hypertension, which are independent risk factors for cardiovascular disease and is known to increase activation of the sympathetic nervous system and cause oxidative stress, endothelial dysfunction, and metabolic dysregulation^2^.

Several studies have shown that chronic hypoxia causes microalbuminuria^3,4^. Microalbuminuria is considered to be a manifestation of diffuse microvascular injury, resulting in secondary kidney damage; microalbuminuria or proteinuria can act as a marker leading to kidney disease^5^. The kidney is sensitive to hypoxia, and intermittent desaturation by sleep apnea is known to cause oxidative stress disorder and damage the kidney^6,7^. Therefore, confirming the association between the STOP-BANG questionnaire for OSA screening and microalbuminuria was meaningful in preventing kidney dysfunction.

Polysomnography (PSG) for OSA confirmation is effective but expensive and has the disadvantage of causing patient discomfort during testing. In contrast, the STOP-BANG questionnaire is inexpensive and does not cause discomfort for patients. The STOP-BANG questionnaire is a useful method for screening patients likely to have OSA, consisting of a simple eight items, and a validation cohort study of preoperative patients confirmed that there is 92.9% sensitivity^8^. This high sensitivity and convenience make it attractive as a screening tool for OSA patients.

Therefore, in this study, we attempted to analyze the relationship between OSA risk and microalbuminuria using Korean National Health and Nutrition Examination Survey (KNHANES) from 2019 to 2020 data for Koreans aged 40 years or older.

## Materials and methods

### Study population

**Figure 1:**
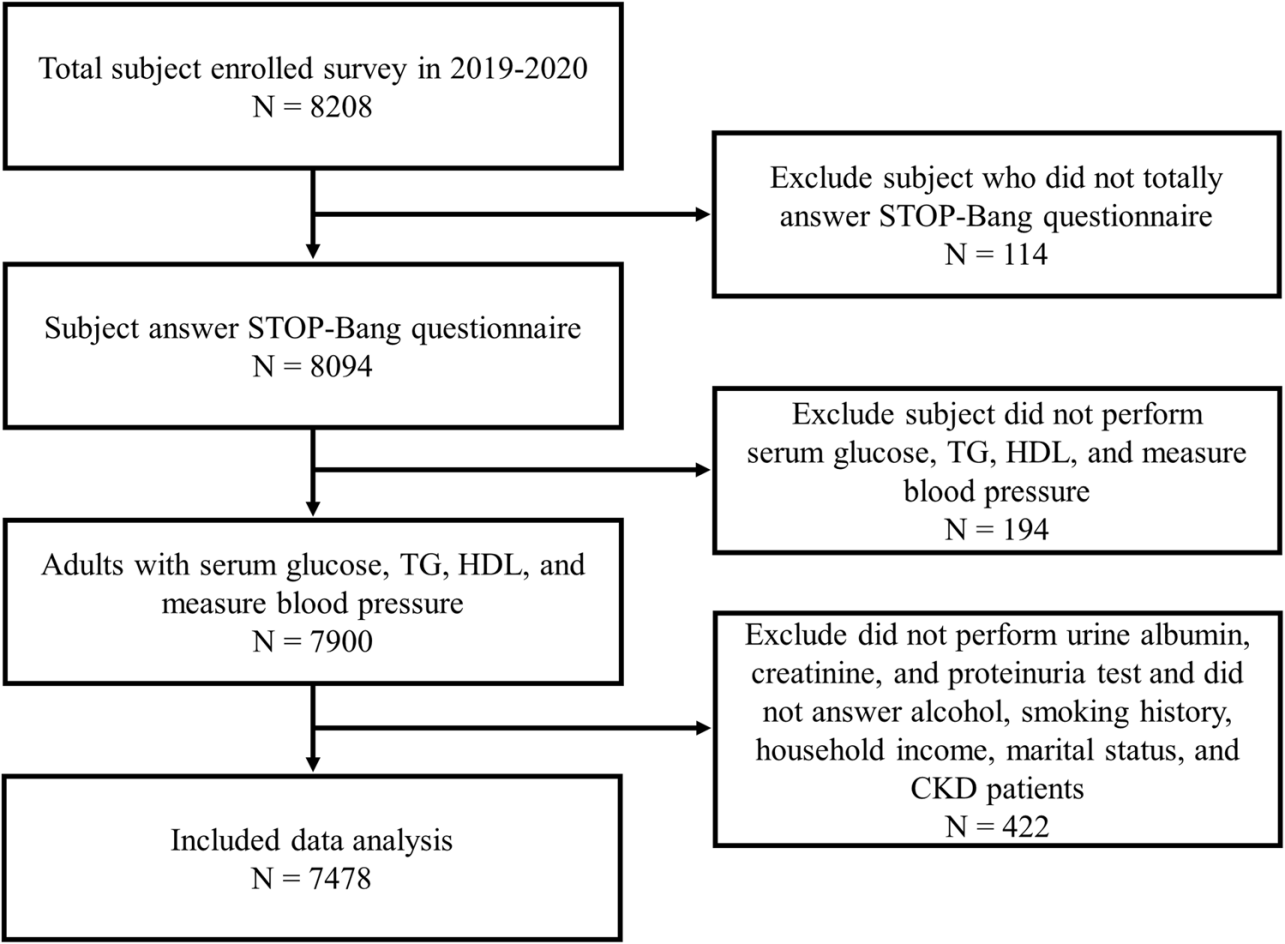
Flowchart of study participants. Among 8208 participants who participated in the Korea National Health and Nutrition Examination Survey from 2019 to 2020, 8094 participants who answered the STOP-BANG questionnaire were included. Finally, a total of 7478 subjects, excluding those who met the exclusion criteria, were analyzed in this study.

Using the 2019 and 2020 KNHANES data, among 8208 adults aged 40 or older who responded to the sleep apnea questionnaire, we analyzed 7478 adults (3289 men and 4189 women) who satisfied the inclusion criteria. The exclusion criteria were as follows: excluding 114 subjects who had no data on STOP-BANG, excluding 194 subjects who had no data on serum glucose, TG (Triglycerides), HDL (high-density lipoprotein), and blood pressure, excluding 422 subjects who had no data on urine albumin, creatinine, proteinuria test, alcohol history, smoking history, household income, marital status, and patients with chronic kidney disease (CKD). Finally, a total of 7478 subjects were analyzed (Fig. 1). All methods and protection of personal information were performed in accordance with the Declaration of Helsinki.

### Measurements of variables

We surveyed the sex, age, smoking history, alcohol history, household income, and educational status of the subjects through questionnaires. We used the STOP-BANG questionnaire for OSA screening. This questionnaire consists of a total of 8 items of four yes/no questions and four clinical characteristics and is as follows: Snoring, Tiredness, Observed apnea during sleep, high blood Pressure, Body Mass Index > 30 kg/m^2^, Age > 50 years, Neck circumference > 40 cm, and male Gender. A score of 3 or more out of 8 was considered a high OSA risk. Less than 2 scores were considered low OSA risk.

Renal function was evaluated by calculating the urinary albumin/creatinine ratio (ACR). We defined microalbuminuria as 30 mg/g ≤ ACR < 300mg/g and macroalbuminuria as ACR > 300 mg/g. The presence of proteinuria was measured using a standard urinary dipstick, and negative (−), trace amount (+ −), one positive (1+), two positives (2+), three positives (3+), four positives (4+), and more than one positive was defined as proteinuria.

### Statistical analysis

We used a t-test and chi-square test to compare the two groups according to the high or low OSA risk. The association between the OSA risk and microalbuminuria according to various factors was analyzed using multivariate logistic regression. The Statistical Package for the Social Sciences statistical software package version 17 (SPSS Inc., Chicago, IL, USA) was used for statistical analysis. In all analyses, the p-value was considered two-tailed, and the statistical significance was set at *p* < 0.05.

### Ethics statement

This research study was conducted retrospectively from data obtained for clinical purposes. The Institutional Review Board of Jeju National University Hospital approved this study.

The Institutional Review Board of Jeju National University Hospital approved that waived the need of informed consent.

## Results

Among 7478 subjects, 2885 were in the high OSA risk group with a score of 3 or higher on the STOP-BANG questionnaire, and 4593 were in the low OSA risk group. In the high OSA risk group, the proportion of men was significantly higher. Age and BMI were also higher than in the low OSA risk group. Systolic/diastolic blood pressure was higher in the high OSA risk group than in the low OSA risk group. Fasting serum glucose and triglycerides were also significantly higher in the high OSA risk group than in the low OSA risk group. The average value of ACR was significantly higher in the high OSA risk group than in the low OSA risk group (36.8 ± 172.2 vs 17.1 ± 82.5; *p* < 0.001) (Table 1).

**Table 1:** Patient demographics according to high or low obstructive sleep apnea risk.

	Obstructive sleep apnea risk		*P* value
	Low (N = 4593)	High (N = 2885)	
	Mean ± SD	Mean ± SD	
Sex			< 0.001
Male	1209 (26.3%)	2080 (72.1%)	
Female	3384 (73.7%)	805 (27.9%)	
Age	57.5 ± 11.6	62.6 ± 10.5	< 0.001
BMI	23.5 ± 2.9	25.5 ± 3.5	< 0.001
HTN	715 (15.6%)	1775 (61.5%)	< 0.001
DM	410 (8.9%)	596 (20.7%)	< 0.001
Hyperlipidemia	969 (21.1%)	1087 (37.7%)	< 0.001
Smoking history	516 (11.2%)	624 (21.6%)	< 0.001
Alcohol history	2944 (64.1%)	2011 (69.7%)	< 0.001
Systolic blood pressure (mmHg)	121.4 ± 17.6	127.7 ± 15.9	< 0.001
Diastolic blood pressure (mmHg)	76.0 ± 9.8	77.9 ± 10.5	< 0.001
Fasting serum glucose (mg/dL)	101.0 ± 21.0	110.4 ± 27.7	< 0.001
Triglycerides (mg/dL)	126.1 ± 96.7	155.2 ± 120.9	< 0.001
HDL (mg/dL)	53.5 ± 12.7	48.0 ± 11.5	< 0.001
ACR (mg/g)	17.7 ± 82.5	36.8 ± 172.2	< 0.001
ACR category			< 0.001
ACR < 30 (mg/g)	4279 (93.2%)	2480 (86.0%)	
30 ≤ ACR < 300 (mg/g)	282 (6.1%)	344 (11.9%)	
ACR > 300 (mg/g)	32 (0.7%)	61 (2.1%)	
Proteinuria	110 (2.4%)	173 (6.0%)	< 0.001
Monthly household income (Unit: US dollar)			< 0.001
< 2000	2425 (52.8%)	1732 (60.0%)	
≥ 2000	2168 (47.2%)	1153 (40.0%)	
Education status			< 0.001
High school or less	2929 (63.8%)	2021 (70.1%)	
College or more	1664 (36.2%)	864 (29.9%)	

*BMI* body mass index, *HTN* hypertension, *DM* diabetes mellitus, *HDL* high-density lipoprotein, *ACR* albumin/creatinine ratio.

**Figure 2:**
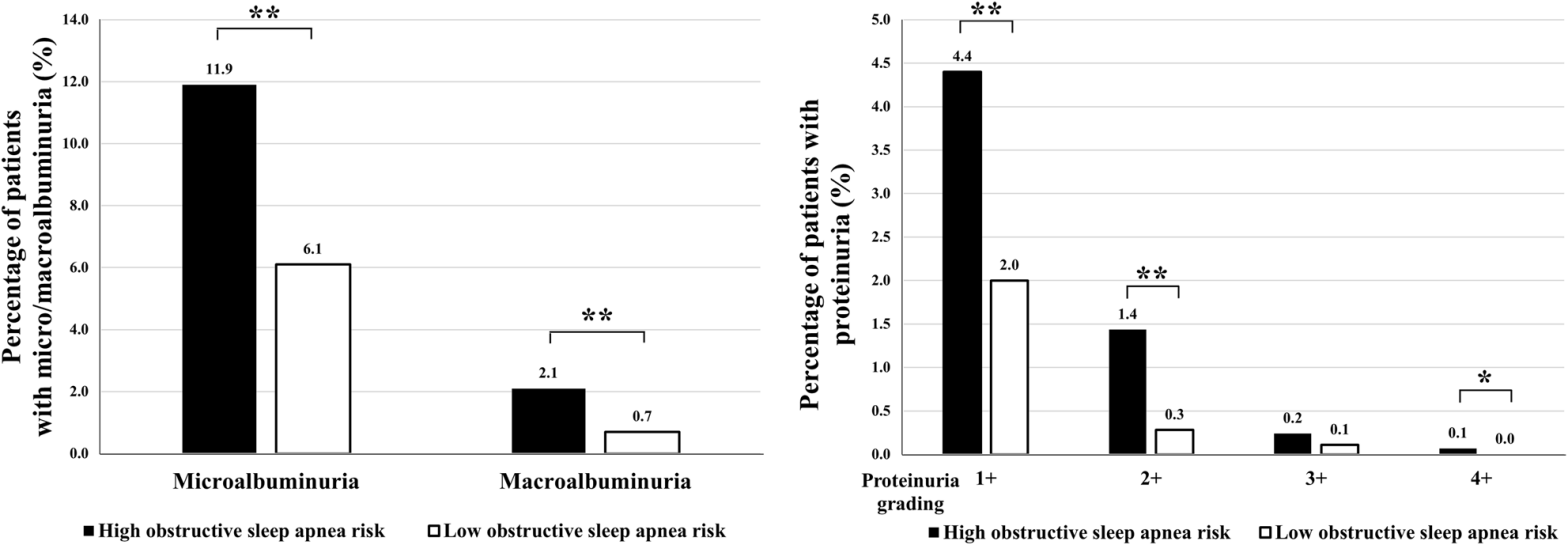
Percentage of patients with microalbuminuria, macroalbuminuria, and proteinuria according to high or low obstructive sleep apnea risk. **p < 0.01, *p < 0.05.

The ratio of microalbuminuria (30 mg/g ≤ ACR < 300 mg/g) and macroalbuminuria (ACR > 300 mg/g) were significantly higher in the high OSA risk group compared to the low OSA risk group (11.9% vs. 6.1%; *p* < 0.001, 2.1% vs. 0.7%; *p* < 0.001). The ratio of proteinuria was significantly higher in the high OSA risk group than in the low OSA risk group when comparing 1+, 2+, and 4+ proteinuria (4.4% vs. 2.0%; *p* < 0.001, 1.4% vs. 0.3%; *p* < 0.001, 0.1% vs 0%; *p* < 0.05) (Fig. 2).

**Figure 3:**
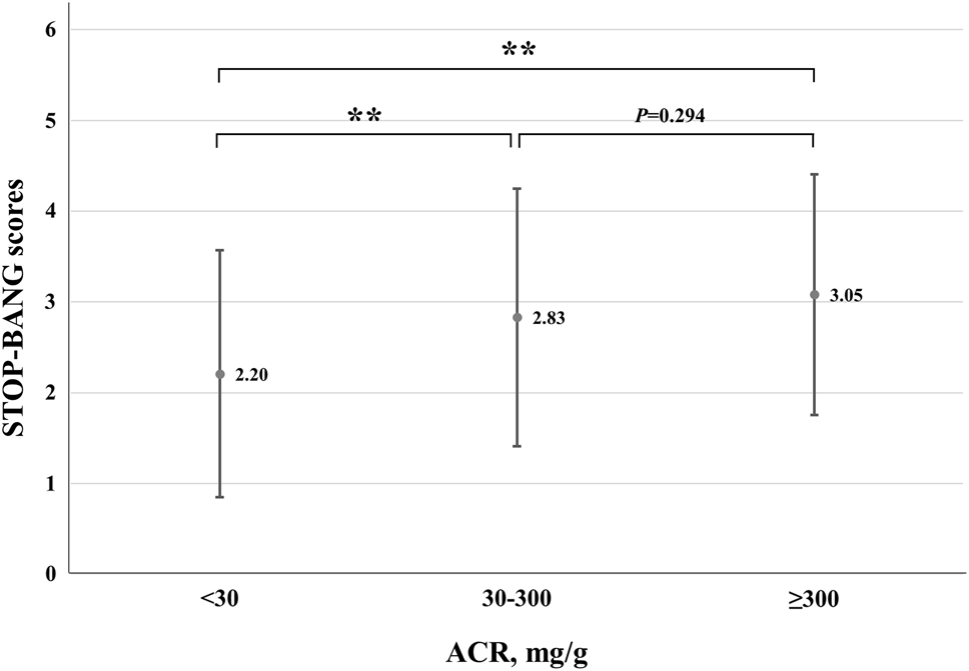
Mean (dot) and standard error (bar) of STOP-BANG scores according to ACR. **p < 0.01.

According to the ACR value, the STOP-BANG score was compared by dividing it into three groups: normal, microalbuminuria, and macroalbuminuria. The average ACR score tended to increase as albuminuria became severe; 2.20 in the normal group, 2.83 in the microalbuminuria group, and 3.05 in the macroalbuminuria group. Compared to the ACR score of the normal group, the ACR score of the microalbuminuria group was significantly higher (2.20 vs. 2.83; *p* < 0.001), and the ACR score of the macroalbuminuria group was also significantly higher (2.20 vs. 3.05; *p* < 0.001), there was no significant correlation between the ACR scores of the microalbuminuria and macroalbuminuria groups (2.83 vs. 3.05; *p* = 0.294) (Fig. 3).

Finally, multivariate logistic regression analysis was conducted between the high and low OSA risk groups, including confounding factors that could affect microalbuminuria, macroalbuminuria, and proteinuria. As a result, it was confirmed that microalbuminuria was significantly correlated in the high OSA risk group than in the low OSA risk group (OR 1.279, 95% CI 1.068–1.532, *p* = 0.008). Macroalbuminuria was significantly correlated in the high OSA risk group than in the low OSA risk group (OR 1.684, 95% CI 1.073–2.530, *p* = 0.022). In addition, proteinuria had a significant correlation in the high OSA risk group than in the low OSA risk group (OR 1.355, 95% CI 1.030–1.783, *p* = 0.030) (Table 2).

**Table 2:** Multivariate logistic regression analysis of renal dysfunction class according to high or low of obstructive sleep apnea risk.

Variable	Model	Odds ratio	95% CI	*p*-value
Microalbuminuria high OSA risk versus low OSA risk	Crude	2.105	1.785–2.482	< 0.001^a^
	Model 1^b^	1.467	1.229–1.751	< 0.001^a^
	Model 2^c^	1.360	1.138–1.626	0.001^a^
	Model 3^d^	1.279	1.068–1.532	0.008^a^
Macroalbuminuria high OSA risk versus low OSA risk	Crude	3.289	2.138–5.059	< 0.001^a^
	Model 1^b^	2.455	1.598–3.740	< 0.001^a^
	Model 2^c^	1.998	1.310–3.047	0.001^a^
	Model 3^d^	1.684	1.073–2.530	0.022^a^
Proteinuria High OSA risk versus Low OSA risk	Crude	2.600	2.037–3.317	< 0.001^a^
	Model 1^b^	1.684	1.282–2.203	< 0.001^a^
	Model 2^c^	1.473	1.123–1.931	0.005^a^
	Model 3^d^	1.355	1.030–1.783	0.030^a^

Abbreviations: CI, confidence interval.

^a^*p* < .05 was considered significantly different.

^b^Model 1: adjusted for sex, age, BMI, smoking, and alcohol consumption.

^c^Model 2: adjusted for sex, age, BMI, smoking, alcohol consumption, serum fasting glucose, triglyceride, high-density lipoprotein cholesterol, family income, and education.

^d^Model 3: adjusted for sex, age, BMI, smoking, alcohol consumption, serum fasting glucose, triglyceride, high-density lipoprotein cholesterol, family income, education, diabetes mellitus, systolic blood pressure, and diastolic blood pressure.

Considering that 20.7% of the high-risk group had diabetes, we performed subgroup analysis according to the presence or absence of diabetes mellitus. As a result, albuminuria/proteinuria had no significant correlation with OSA risk (Supplementary Table S1). However, in the group without diabetes mellitus, albuminuria/proteinuria was significantly correlated with the high OSA risk group than with the low OSA risk group (Supplementary Table S2).

## Discussion

This study analyzed the relationship between OSA risk and microalbuminuria in Koreans aged 40 and above. As a result, the average value of ACR was higher in the high OSA risk group than in the low OSA risk group, and it was confirmed that there was a significant correlation between microalbuminuria and the high OSA risk. Also, there was a significant association between the OSA risk and macroalbuminuria/proteinuria.

Albuminuria is a marker of renal damage in the early stages of CKD^9^. Microalbuminuria and OSA are known to be caused by similar pathogenetic mechanisms, and OSA has been demonstrated to be independently associated with albuminuria^9,10^. A study that conducted polysomnography and urine collection in 496 OSA patients reported that the higher the severity of OSA, the higher the microalbuminuria^11^. In addition, a study of 507 sleep-disordered breathing elderly reported that the increased respiratory disturbance index and nocturnal hypoxemia were associated with high ACR^12^.

The pathological mechanisms affecting kidney function in OSA patients are not known precisely. However, several possible mechanisms have been suggested. First, hypoxemia caused by sleep apnea was significantly related. This hypoxemia can affect renal tissue hypoxia and induce tubulointerstitial injury and renal vasculature damage^13^. The second is due to the high correlation between OSA and blood pressure. In patients with OSA, not only is the prevalence of hypertension high, but the risk of incident hypertension also increases gradually. Therefore, it is also connected to the renin–angiotensin–aldosterone system (RAAS) activation and endothelial dysfunction^14^. The third is that kidney function is affected by the increased sympathetic activity in patients with OSA. Kidney function is aggravated by sympathetic hyperactivity, and renal structural damage may occur^15^. Finally, it is due to oxidative stress caused by OSA. The unbalanced production of reactive oxygen species (ROS) causes oxidative stress, affecting the kidney's structural and functional changes^16^.

Several studies show that renal function affects OSA. It has been reported that sleep apnea occurs in at least 50% to 60% of CKD patients^17^, and there is also a report that CKD patients show ten times higher sleep apnea prevalence than the general population^18^. Although the exact pathological mechanism of kidney disease-induced OSA has not been fully elucidated, several possible mechanisms exist. The first is that altered chemoreflex responsiveness in CKD patients can affect the respiratory system and respiratory instability, contributing to the occurrence of OSA^19^. The second is that pharyngeal narrowing can affect the occurrence of OSA. It has been reported that ESRD (End Stage Renal Disease) patients have a relatively narrow pharyngeal area compared to subjects with normal renal function^20^, and uremic myopathy or neuropathy can directly affect the upper airway dilator muscle, resulting in a decrease in airway size^21^.

We analyzed the STOP-BANG questionnaire by setting the standard for the BMI item to exceed 30 rather than the previous 35. This is because in the Asian population, obese patients with a BMI of over 35 are relatively few compared to Western people, and a BMI cutoff of 30 was suggested in the STOP-BANG questionnaire study for OSA patients targeting the Asian population^22^. The STOP-BANG questionnaire is useful as a simple screening tool for OSA and shows the convenience of use and excellent sensitivity. In addition, the STOP-BANG questionnaire showed a moderately high level of sensitivity and specificity in surgical patients and a slightly higher sensitivity in patients with moderate to severe OSA^8^.

A strength of this study is that a relatively large number of adult participants who can represent Koreans were included in the analysis. In addition, urine ACR was measured to confirm that the incidence of micro/macroalbuminuria was significantly related to patients with a high possibility of OSA. Also proteinuria had significant correlation with the patients with a high possibility of OSA. The STOP-BANG survey has the drawback of low specificity but can measure the risk of OSA with high sensitivity. Therefore, it is effective in screening for the risk of OSA, enabling the prediction of both OSA risk and renal function risk without requiring significant cost and time. Despite these strengths, this study had several limitations. First, the evaluation of OSA was evaluated using a questionnaire, and an objective test, such as PSG, was not used. In addition, the STOP-BANG questionnaire tends to have high sensitivity but low specificity. Therefore, caution is needed in interpreting such correlations. Moreover, for individuals identified as high risk for OSA in the STOP-BANG survey, careful attention to renal function may be warranted. Also, further study in objectively proven OSA patients is needed. Second, since this study was cross-sectional, proving a high correlation between albuminuria/proteinuria and OSA is possible, but it is difficult to know the causal relationship. Third, several possible pathophysiologic mechanisms can be proposed for this association, but the exact mechanism still needs to be explained. Therefore, additional prospective studies on these causal relationships and exact mechanisms will be needed.

In conclusion, this study confirmed a significant association between high OSA risk and albuminuria/proteinuria in Koreans aged 40 years and above. Therefore, renal function evaluation is required in high OSA risk patients, and OSA diagnosis through PSG test and treatment is necessary.

### Supplementary Information

Supplementary Information 1.

Supplementary Information 2.

## Data Availability

All available data generated or analyzed during this study are included in this published article. Other raw data are not available because of regulation of data sharing in the Republic of Korea. Contact (Suk Won Chang, swjang37111@gmail.com) if someone wants to request the data from this study.

## Abbreviations

OSA: Obstructive sleep apnea
ACR: Albumin/creatinine ratio
CI: Confidence interval
OR: Odds ratio
PSG: Polysomnography
KNHANES: Korean national health and nutrition examination survey
TG: Triglycerides
HDL: High-density lipoprotein
CKD: Chronic kidney disease
RAAS: Renin-angiotensin-aldosterone system
ROS: Reactive oxygen species
ESRD: End stage renal disease
