# Supplementary material for: Increasing obstructive sleep apnea risk is associated with albuminuria in Korean adults: cross-sectional analysis
Source: Sci Rep. 2024 Mar 20;14:6676. doi: 10.1038/s41598-024-57394-3 (PMC10954636; doi:10.1038/s41598-024-57394-3)
Supplement: Supplementary file 1 — Supplementary Information 1. [file 41598_2024_57394_MOESM1_ESM.docx]

Supplementary table 1. Multivariate logistic regression analysis of renal dysfunction class according to high or low of obstructive sleep apnea risk in diabetes mellitus subjects

| Variable | Model | Odds ratio | 95% CI | *p*-value |
| --- | --- | --- | --- | --- |
| Microalbuminuria  High OSA risk versus Low OSA risk | Crude  Model 1^b^  Model 2^c^  Model 3^d^ | 1.248  1.043  1.161  1.033 | 0.906-1.720  0.710-1.602  0.787-1.713  0.694-1.538 | 0.176  0.634  0.452  0.872 |
| Macroalbuminuria  High OSA risk versus Low OSA risk | Crude | 1.605 | 0.881-2.925 | 1.122 |
|  | Model 1^b^ | 1.202 | 0.597-2.420 | 0.607 |
|  | Model 2^c^ | 1.259 | 0.617-2.570 | 0.526 |
|  | Model 3^d^ | 1.055 | 0.504-2.209 | 0.887 |
| Proteinuria  High OSA risk versus Low OSA risk | Crude  Model 1^b^  Model 2^c^  Model 3^d^ | 1.692  1.078  1.148  1.056 | 1.076-2.662  0.634-1.831  0.669-1.969  0.610-1.830 | 0.023^a^  0.782  0.617  0.845 |

Abbreviations: CI, confidence interval.

^a^*p* < .05 was considered significantly different.

^b^Model 1: adjusted for sex, age, BMI, smoking, and alcohol consumption.

^c^Model 2: adjusted for sex, age, BMI, smoking, alcohol consumption, serum fasting glucose, triglyceride, high-density lipoprotein cholesterol, family income, and education.

^d^Model 3: adjusted for sex, age, BMI, smoking, alcohol consumption, serum fasting glucose, triglyceride, high-density lipoprotein cholesterol, family income, education, systolic blood pressure, and diastolic blood pressure.
