# Supplementary material for: Increasing obstructive sleep apnea risk is associated with albuminuria in Korean adults: cross-sectional analysis
Source: Sci Rep. 2024 Mar 20;14:6676. doi: 10.1038/s41598-024-57394-3 (PMC10954636; doi:10.1038/s41598-024-57394-3)
Supplement: Supplementary file 2 — Supplementary Information 2. [file 41598_2024_57394_MOESM2_ESM.docx]

Supplementary table 2. Multivariate logistic regression analysis of renal dysfunction class according to high or low of obstructive sleep apnea risk in non-diabetes mellitus subjects

| Variable | Model | Odds ratio | 95% CI | *p*-value |
| --- | --- | --- | --- | --- |
| Microalbuminuria  High OSA risk versus Low OSA risk | Crude  Model 1^b^  Model 2^c^  Model 3^d^ | 2.022  1.478  1.475  1.323 | 1.666-2.455  1.203-1.817  1.166-1.865  1.073-1.632 | <0.001^a^  <0.001^a^  0.001^a^  0.009^a^ |
| Macroalbuminuria  High OSA risk versus Low OSA risk | Crude | 2.899 | 1.628-5.162 | <0.001^a^ |
|  | Model 1^b^ | 2.377 | 1.324-4.269 | 0.004^a^ |
|  | Model 2^c^ | 2.377 | 1.324-4.269 | 0.004^a^ |
|  | Model 3^d^ | 2.243 | 1.251-4.022 | 0.007^a^ |
| Proteinuria  High OSA risk versus Low OSA risk | Crude  Model 1^b^  Model 2^c^  Model 3^d^ | 2.532  1.752  1.570  1.548 | 1.907-3.363  1.268-2.422  1.134-2.173  1.123-2.133 | <0.001^a^  0.001^a^  0.007^a^  0.008^a^ |

Abbreviations: CI, confidence interval.

^a^*p* < .05 was considered significantly different.

^b^Model 1: adjusted for sex, age, BMI, smoking, and alcohol consumption.

^c^Model 2: adjusted for sex, age, BMI, smoking, alcohol consumption, serum fasting glucose, triglyceride, high-density lipoprotein cholesterol, family income, and education.

^d^Model 3: adjusted for sex, age, BMI, smoking, alcohol consumption, serum fasting glucose, triglyceride, high-density lipoprotein cholesterol, family income, education, systolic blood pressure, and diastolic blood pressure.
